# Why Meaning in Life Matters for Societal Flourishing

**DOI:** 10.3389/fpsyg.2020.601899

**Published:** 2021-01-14

**Authors:** Clay Routledge, Taylor A. FioRito

**Affiliations:** ^1^Department of Management and Marketing, North Dakota State University, Fargo, ND, United States; ^2^Department of Psychology, North Dakota State University, Fargo, ND, United States

**Keywords:** meaning, flourishing, motivation, economics, prosocial behavior, self-regulation

## Introduction

Meaning in life reflects the feeling that one's existence has significance, purpose, and coherence (see Heintzelman and King, 2014). A growing body of research identifies meaning in life as a fundamental human need that strongly influences both psychological and physical well-being (see Vail and Routledge, 2020). Individuals who perceive their lives as full of meaning live longer, healthier, and happier lives than those less inclined to view their lives as meaningful. Despite the growing recognition that meaning in life is vital for humans, scholars have largely ignored how meaning influences broader societal flourishing. We propose that meaning has important social and economic implications, particularly when societies are facing major existential threats such as the current COVID-19 pandemic. More specifically, we argue that meaning functions as a self-regulatory and motivational intrapsychic resource that orients people toward the types of cognitions and behaviors that build and sustain healthy communities and societies.

## Meaning Promotes Psychological and Physical Health

Scholars have long recognized that meaning in life is an important psychological need. The more people feel meaningful, the more they experience overall positive psychological well-being (e.g., Steger and Frazier, 2005). Moreover, meaning reduces the risk for depression (e.g., Disabato et al., 2017), addiction (e.g., Kinnier et al., 1994), and suicide (e.g., Edwards and Holden, 2001). Meaning is also positively associated with physical health and longevity (e.g., Czekierda et al., 2017).

## A Motivational View of Meaning

Understanding why meaning matters so much for health and well-being paves the way for a broader analysis of existential health and the role it plays in societal flourishing. Meaning positively contributes to psychological and physical health because of its motivational and self-regulatory nature (see Hooker et al., 2018; Routledge, 2018). For example, meaning in life, but not well-being indicators such as positive affect or optimism, positively predicts physical activity (Hooker and Masters, 2016) and when people are thinking about what gives their lives meaning, they are more likely to engage in physical exercise and to exercise for longer intervals, even if they were previously physically inactive (Hooker and Masters, 2018).

More broadly, meaning drives goal pursuit (see Routledge, 2018). For instance, when individuals bring to mind and reflect on meaningful life memories, they subsequently report greater perceptions of meaning and motivation to pursue goals, and this motivational effect cannot be attributed to positive affect (Sedikides and Wildschut, 2018). Findings such as these reveal that when people are focused on what gives their lives meaning, they are generally more agentic and inspired. These and related findings (see Steger et al., 2006) also highlight that meaning isn't synonymous with other well-being indicators.

Research identifying meaning as a coping resource further reveals its motivational nature (see Park, 2010). For example, when people face mental health challenges, meaning in life may play a vital role in treatment success by motivating people to be compliant and actively engaged in the treatment process. Indeed, people with greater perceptions of meaning respond more positively to psychotherapy (Debats, 1996). Life often involves experiences of uncertainty, stress, sadness, and loss. Eventually, we all lose loved ones and must face death ourselves. Critically, meaning is a vital psychological resource for coping with these challenges (Park and Folkman, 1997). Those who are able to respond to tragedy and loss in ways that affirm meaning are better able to move forward with their lives in productive ways and to be at peace with their own mortality (see Routledge and Vess, 2018). When people believe their lives matter, they have a reason to regulate their behavior in ways that helps keep them alive and thriving.

## Meaning as a Critical Ingredient of Societal Flourishing

The well-being of any society is directly linked to the well-being of the individuals living in it. Thus, the fact that meaning in life supports individual flourishing provides critical evidence that it also promotes societal flourishing. However, the positive influence of meaning on societal well-being is more than the sum of individuals regulating their own behavior in ways that help them stay healthy and pursue self-focused goals. Meaning in life and the agency it generates has important implications for social and economic health, which are two critical ingredients of societal flourishing.

Meaning promotes social and community engagement. Numerous studies have identified social bonds as a primary source of meaning in life. For example, when asked to detail in writing what gives their lives meaning, the most frequently reported source of meaning is close relationships (Nelson et al., 2019). However, research also indicates that meaning promotes the pursuit of social connections. For example, Stavrova and Luhmann (2016) observed that meaning in life positively predicted the extent to which individuals felt connected to their community, family, friends, and spouse/partner 10 years later. In a second study, these researchers found even stronger evidence for a social motivational function of meaning; higher levels of meaning predicted a greater likelihood of future participation in voluntary associations, and, among single people, a greater likelihood of getting married. Such findings are consistent with laboratory research showing that when people reflect on personally meaningful past social experiences, they become more motivated to pursue social goals and more confident that they can overcome problems in their relationships (Abeyta et al., 2015).

Moreover, the more individuals report a desire to live a meaningful life, the more they engage in prosocial behavior such as volunteering and charitable giving (FioRito et al., in press). This suggests that the need for meaning orients people toward helping others and supporting the social organizations that they believe improve society.

Meaning in life may also promote societal flourishing at the economic level. For example, a sense of purpose in life predicts gains in household income and net worth over time (Hill et al., 2016). Since meaning promotes self-control and goal-directed behavior generally, it likely supports the types of economic decision-making and work-related goals that lead to greater financial security. Greater financial security is key to the economic health of communities and the global economy. And individuals are better positioned to support other important features of community life (e.g., the arts) and to help those in need with charitable giving when they are able to meet their own financial needs.

## Using Meaning to Overcome Major Societal Threats and Challenges

Since meaning in life is a resource the helps people cope with stress, uncertainty, anxiety, and trauma it may also play a vital role in helping communities and the broader society face collective threats and challenges. Recent studies, for instance, found that higher meaning in life is associated with lower levels of anxiety and COVID-19 stress (Trzebinski et al., 2020) and lower levels of mental distress among those facing pandemic-related stress (Schnell and Krampe, 2020). In order to successfully respond to and recover from collective threats such as pandemics, economic recessions, and natural disasters, humans need to possess the psychological fortitude that not only helps them manage their personal anxieties, but that also drives them to want to positively contribute to the world around them.

## Toward a More Outward Focused Existential Psychology

Much of the research in existential psychology has focused on how meaning contributes to individual health and well-being and has ignored many of the ways that meaning might orient people outward. In the current analysis, by focusing on the self-regulatory and motivational functions meaning serves and connecting those functions to outcomes beyond the individual, we hope to inspire more research directed toward exploring the vital role meaning may play in promoting societal flourishing. We propose that when people view their lives as meaningful, they are better positioned and more motivated to take care of themselves and make valuable contributions to their families, communities, nation, and the world.

## Author Contributions

All authors listed have made a substantial, direct and intellectual contribution to the work, and approved it for publication.

## Conflict of Interest

The authors declare that the research was conducted in the absence of any commercial or financial relationships that could be construed as a potential conflict of interest.
